# Effectiveness of curriculum-based sexual and reproductive health education on healthy sexual behaviors among year one students at Arba Minch University: A quasi-experimental study

**DOI:** 10.1371/journal.pone.0288582

**Published:** 2023-10-31

**Authors:** Negussie Boti Sidamo, Sultan Hussen, Mulugeta Shegaze Shimbre, Eshetu Zerihun, Wanzahun Godana Boynito, Sintayehu Abebe, Tamiru Shibiru, Simon Shibiru, Woyinshet Gebretsadik, Nathan Desalegn, Bilcha Oumer, Gebremaryam Temesgen Birgoda, Hanan Abdulkadir

**Affiliations:** 1 Arba Minch University, College of Medicine & Health Sciences, School of Public Health, Arba Minch, Ethiopia; 2 School of Public Health, Cheeloo College of Medicine, Shandong University, Jinan, Shandong, 250012, China; 3 Arba Minch University, College of Medicine & Health Sciences, School of Medicine, Arba Minch, Ethiopia; 4 Department of Biology, Arba Minch University, College of Natural Sciences, Arba Minch, Ethiopia; 5 Arba Minch University, College of Medicine & Health Sciences, School of Nursing, Arba Minch, Ethiopia; 6 Department of Midwifery, Arba Minch Health sciences college, Arba Minch, Ethiopia; 7 Department of Midwifery, Arba Minch University, College of Medicine and Health Sciences, Arba Minch, Ethiopia; Norwegian Refugee Council, JORDAN

## Abstract

**Introduction:**

Curriculum-based sexual and reproductive health education (CBSRHE) is one of the preventive strategies targeting youth in higher institutions to protect them from sexual and reproductive health problems, despite never assessing the effect in Ethiopia. Therefore, this study aimed to assess the effect of CBSRHE on knowledge and attitude about SRH services to have safer sexual behaviors among first-year students of Arba Minch University.

**Method:**

We conducted a quasi-experimental study among purposively selected campuses. The campuses were allocated to (i) an intervention arm consisting of curriculum-based sexual and reproductive health, or (ii) a control arm for those who were free from intervention. Data was collected, at baseline and immediately after the intervention ended by using a structured self-administered questionnaire. To compare differences in the change from baseline to post-intervention between the two arms we use the chi-square test and independent-samples t-test. To see the effect of the CBSRHE by controlling the effect confounding inverse probability-weighted analysis was conducted.

**Result:**

A total of 832 and 820 students participated in a baseline and post-test respectively. The proportion of youth who practice receptive penetrative sex decreases from 40.9% to 28.3% in the intervention arm compared to 37.6% to 37.3% in the non-intervention arm between baseline and end line, with statistically significant differences between groups. However, there are statistically significant differences between the intervention and control groups in terms of changes in contraception utilization (X^2^ = 1.21; P>0.05). Furthermore, there were significant improvements in knowledge and attitude among the intervention arm a comprehensive knowledge of HIV/AIDS (ATE = 0.22,95% CI, 0.14 to 0.29; p < 0.01), an average change of attitude toward HIV/AIDS(ATE = 1.32, 95% CI, 1.18 to 1.47; p < 0.01), comprehensive condom knowledge score (ATE = 0.23, 95% CI, 0.13 to 0.33; p < 0.01) and the average change of attitude toward condom score (ATE = 1.83, 95% CI, 1.19 to 2.77; p < 0.01).

**Conclusion and recommendation:**

It was found that there was a significant difference in knowledge and attitude toward a disk sexual behaviors among r one student. This implies that the educational authority o the country can gain through the adoption of courses to all universities across the country, besides doing further comparative studies to determine the long-term effect of the course supported with models and/or theories like the theory of change.

## Introduction

Worldwide, nearly two billion people are in the transitional years, ages 10 to 24, of these 70% are in developing nations and these youth account for 20% of new HIV infection [1,2]. About 70% of these infections occur in sub-Saharan Africa (SSA) [1]. Condomless receptive vagial sex is one of the public health problems among young people globally [3]. Evidence shows that condomless receptive vaginal/anal sex was one of the leading risk factors for sexually transmitted infections (STIs), Human Immune Virus (HIV) infection, and unintended pregnancies [3–6]. Multiple vaginal/ anal sex partners are affected by cultural values and public health policies that are different in each country and setting [3]. In Ethiopia, youths are estimated to contribute to 26% of new HIV infections [7].

In Ethiopia, higher education institutions host youths aged between 19–24 years and they are the largest segments of the population [2]. Studies conducted in different parts of Ethiopia reveal that 45% to 65.6% of youths had sufficient knowledge of condom utilization and HIV/AIDS receptively [8,9]. However, only 38.3% of youth ever used a condom and 46.79% of respondents had an interest in using condoms and the rest had no intention [10,11]. Ethiopia is among the highest unmet needs for family planning ranging from 16.2% up to 30.9% [12,13]. Moreover, in Ethiopia, early marriage has high rates of 44.8% with a minimum of 9 years and maximum ages of 23 years old [14]

Due to the increase in condomless receptive vaginal/anal sex among youths governmental and non-government organizations use different approaches to promote better healthy sexual outcomes among youths [15]. Among those approaches, Curriculum Based Sexual and Reproductive Health Education (CBSRHE) has great potential for providing the knowledge and practical skills to make safe and informed choices with regard to their sexual and reproductive life [16,17].

CBSRHE is defined as a program in schools, community agencies, health facilities, and other settings where young people can accumulate regularly that help youth to have a satisfying safe sexual life, the ability to reproduce, and the freedom to decide the time, frequency, and else [8,18]. Studies conducted in different parts of the world show that CBSRHE has great potential to provide young people with the necessary information about their bodies and sexuality, reduce engagement in risky sexual behavior, reduce misinformation, shame, and anxiety, and improve their abilities to make safe and informed choices about their sexual and reproductive health [15–17].

Different countries started the implementation of CBSRHE at different time [15,17,19]. However, the Ethiopian government started the implementation of curriculum-based sexual and reproductive health education in 2016 in Ethiopian Universities to improve youth’s knowledge and practical skills to make safe and informed choices with regard to their sexual and reproductive lives of youth during their stay at university [8]. Therefore, the aim of this study was to assess the short-term effect of CBSRHE on knowledge and attitude about SRH services among first-year students of Arba Minch University.

## Methods

### Study design and setting

Arba Minch University is a residential national university in Arba Minch, Southern, Ethiopia. It is about 435 kilometers south of Addis Ababa. This university was among the first generation university in Ethiopia. The University is sited on five campuses based around Arba Minch town and one campus in Sawla town, of which are urban. Teaching-learning, research, and community service are the paradigms of the university. In 2016/17, the University’s intake capacity is more than 32,977 students (23, 092 males and 9885 females) in regular and extension programs on six campuses situated across university [20]. A quasi-experimental study was conducted to meet the objective of the study. The data from study participants was collected in two phases. The baseline data was collected in October 2017 and the end-term data was collected in February 2018.

### Population

In Ethiopia, the minimum age to join a university education is 19 years. All year one regular undergraduate students (students admitted from grade 12) of Arba Minch University were the target for the study and those students in selected departments were the study population. All first-year students who were attending the regular programs during the intervention period were considered for the study. Those students previously exposed to CBSRHE and those who were unable to respond as result of illness were excluded.

### Sampling size determination and sampling technique

The number of students for the study was determined by Open Statcalc based on the following assumptions: proportion of condom use among controls and intervention groups 59.1% and 71.4% respectively [21], 95% confidence interval, 80% power with a minimum detectable alternative of ± 5%. Accordingly, the calculated sample size was 504 participants. Assuming a study loss to follow up of 10% and a design effect of 1.5, the total minimum sample size needed for this study was 504* 0.1+ 504 = 554*1.5 = 832 students. The sampling procedure applied was cluster sampling and the sampling frame was obtained from the Arba Minch University registrar’s office. Arba Minch University has campuses situated in Arba Minch town and Sawla town where we took intervention and control groups respectively. We purposively selected 8 departments from each group for the study excluding those departments that previously taken CBSRHE. For each selected department we have assigned a proportional number of participants from the cluster and the participants are selected randomly from the departments by a computer-generated random table. Eight hundred Thirty-Two (416 from each group) were selected for the study.

### Summary of intervention

The intervention (CBSRHE) is a common course designed to provide for all year one students in semester wise manner. The total load of the course is 3 credit hours/week for 16 weeks and the course is delivered by Arba Minch University College of Medicine and health science staff that are specialized in Reproductive health, Maternal and Reproductive health nursing, Clinical Midwifery, and General Public health. The course is delivered in three chapters each labeled as Basics of HIV/AIDS, Sexual and Reproductive Health, and Gender and gender-based violence. The education was delivered using brainstorming, lectures, Case studies, discussion, role play, and demonstration. All the participants in the intervention arm were involved in all sessions and the delivery was in English language.

### Outcome measures

#### SRH knowledge

To assess SRH knowledge we use knowledge about HIV/AIDS, knowledge about contraceptive methods, and knowledge about a condom because they are commonly used measurements of knowledge of HIV/STIs and pregnancy prevention studies. To assess students’ comprehensive knowledge about HIV/AIDS was measured using the five-item scale question which has been used and validated in HIV indicator surveys and demographic and health survey [22–24]. The scale was comprised of the question like, Knowing that consistent condom use during sexual intercourse and having just one uninfected faithful partner can reduce the chances of contracting HIV, knowing that a healthy-looking person can have HIV, and correctly rejecting the two most common local misconceptions about HIV transmission or prevention. (Such misconceptions usually include "AIDS transmitted by mosquito bites," and "A person can become infected by sharing food with a person who has AIDS"). This variable will be treated as continuous and each variable coded as "1" or comprehensive knowledge about HIV/AIDS", If a participant answered all five questions correctly and "0" if the participant missed at least one question. To assess students’ knowledge of condoms, we develop four items questions developed after reviewing a similar study. The scale has composed of variables like having heard about the male and female condoms; condoms are an effective method to prevent unwanted pregnancy, HIV/AIDS, and STDs. The right response was coded as "1" and the wrong answer as "0" [16]. To assess respondent knowledge about contraceptive methods was measured by using the following question, "Have you ever heard about contraceptives?" Affirmative responses (yes) led to subsequent questions on the reasons for use of contraceptives, methods, and the sources known to her. Then the respondent was considered knowledgeable if they correctly provided responses based on the listed options on the tool. This variable will be treated as continuous and each variable coded as "1” or comprehensive knowledge about contraceptive methods**”,** If a participant answered all five questions correctly and “0” if the participant missed at least one [25,26].

#### SRH attitudes

SRH attitudes were measured based on attitudes to HIV/STIs pregnancy prevention and attitudes toward gender-equitable norms [27]. To assess students’ attitudes towards HIV/AIDS patients were measured using ten Likert scale item questions. Then the sum of the scores for individual respondents was then calculated, and the mean of all the scores was thereafter calculated [24,28]. This variable will be treated as continuous and each variable coded as "1” or have a positive attitude if the respondent scored up to or above the mean and “0” or have a negative attitude if the respondent scored below the mean. To assess students’ attitudes towards condoms were measured using thirty Likert scale items developed after reviewing previously done pieces of literature [26,29]. Respondents were presented with the following statements. Response categories included: 1 = agree, 2 = not sure, and 3 = disagree. To assess respondent attitudes toward gender-equitable norms were measured by 24 items questions adapted from Ethiopian standard gender attitudinal scales^6^. Attitudes towards the concept of gender equality were measured with four domain items, Violence domain items, sexual relationships domain items, reproductive health and disease preventions domain items, and daily life domain items were inquired. Each of the above items was scored on a 3-point scale, where 1 = agree, 2 = partially agree, and 3 = do not agree. High scores represent high support for gender-equitable norms. Certain items were reverse-scored if a high score would reflect low support for gender equity. Responses to each item were summed^6^. This variable will be treated as continuous and each variable coded as "1" or" Have a positive attitude towards gender-equitable norms" if the student scores high and “0” if the student scores low.

#### Sexual behavior

To assess students’ sexual behavior we used a five-item scale question, which is adapted from Youth Risk Behavior Surveillance 2017 tool [30]. The scale is comprised of the following question, Have you ever had sexual intercourse without a condom? During your life, with how many people have you had sexual intercourse? During the past 3 months, with how many people did you have sexual intercourse? The last time you had sexual intercourse, did you or your partner use a condom? The last time you had sexual intercourse, what one method did you or your partner use to prevent pregnancy? Sexual behavior coded as "1" or "Have risky sexual behavior if the participant engaged in at least one of the above behaviors and "0" if the participant did not engage in any of the above behavior in the last 3 months

### Data collection procedures and instruments

Data were collected using a self-administered survey prepared on a paper questionnaire. The questionnaire was initially adopted from the WHO knowledge, attitudes, beliefs, and practices survey instrument, and YRBS instrument, and further modified based on available literature [26–28,30,31]. The collection tool has three sections socio-demographic characteristics, sexual behavior-related questions, knowledge, attitude, and practice questions. The baseline data were collected from study participants before starting CBSRHE. The unique confidential identification number was assigned to each student to allow for matching their responses across time points. After the collection of baseline data, the intervention group starts comprehensive sexual and reproductive health intervention for sixteen-week to equip students with the knowledge, skills, and attitudes needed to prevent themselves from sexual reproductive health problems and bring positive behavioral changes on sexual and reproductive issues. The education was delivered using brainstorming, lectures, case studies, discussion, and demonstration methods by reproductive health specialized instructors from the College of Medicine and Health Science and Psychology department. Immediately, after the last education session before the final exam period, the post-intervention stage, the same questionnaire that was used in the pre-intervention stage was administered to the same students who were selected at the pre-intervention stage in both the intervention and the control groups. To maximize the validity of these data, the research team emphasized that: (*a*) all individual responses were confidential and would not be revealed to others and (*b*) all students should not discuss any of the questions with other students (c) they were encouraged to answer all questions, with emphasis placed on the importance of honest answers. Furthermore, to reduce contamination, campuses for the experimental group were recruited from a different geographical region than the campus for the control group. Six male and six female data collectors were participated in the data collection and were supervised by five research team members. The data were collected at both phases with paper printed questionnaires distributed for self administer.

### Data quality control

For maintaining data quality different technique was used first training was given for data collectors and supervisors. The research team, as well as data supervisors, conducted a day-to-day follow-up during data collection. Pre-testing was done at Arba Minch Health Science College with 5% of the study participants slight correction was done on the tool. The data were cleaned double entered and cross-checked for their completeness and link to the unique identification number before analysis.

### Data processing and analysis

The completeness and consistency of the data were checked, coded, and English version of the collected data was double entered in computer software Epi-data 3.1 and exported to STATA version 14.0 statistical software for further analysis. Descriptive statistics were done using measures of central tendency (mean, median) and measures of dispersion (range, standard deviation), as appropriate to present the students’ mean scores on knowledge, attitudes, and behavior before and after the intervention. To compare categorical outcome variables before and after intervention as well as between the intervention and control group Pearson chi-squared test was conducted. Furthermore, to compare continuous outcome variables before and after the intervention was tested by paired t-test. We estimate the intervention effect by subtracting the baseline from the estimates at the end line and then calculating the difference between the comparison and intervention sub-counties (the difference-indifference analysis). The difference-in-differences analysis assumes a common trend in the outcome in both the intervention and comparison areas. We measure potential confounders at baseline and end line and adjust our analysis for any compositional changes over time in these confounders by using inverse probability-weighted analyses. All statistical analyses will be considered statistically significant for p-values less than 0.05.

## Results

### Socio-demographic characteristics of the study participants

Four hundred sixteen students were involved in the pre-intervention for each group, and 400 and 404 students were involved among the intervention and control groups respectively resulted in a response rate of 96.75%. After intervention Four hundred Eleven and Four hundred nine questionnaires were distributed among the groups. Among those 396 and 397, students were participated giving that a response rate of 96.71% [Table 1].

**Table 1:** Socio-demographic characteristics of the study participants in Arba Minch University, Ethiopia, 2018/19.

Study group	Variables	Subcategories	Pre-Intervention N (%)	Post- Intervention N (%)	P-value
**(Intervention Group)**	Sex	Male	297(74.1)	294(74.2)	*X*^*2 =*^ *0*.*206* *P = 0*.*694*
		Female	104(25.9)	102(25.8)	
	Age in year	Mean ± SD	19.55 ±1.94	19.60±1.14	
	Religion	Orthodox	259(64.6)	256(64.6)	*X*^*2 =*^ *7*.*39* *P = 0*.*807*
		Catholic	2(0.5)	2(0.5)	
		Protestant	97(24.2)	95(24.0)	
		Muslim	39(9.7)	38(9.6)	
		Others	4(1.0)	5(1.3)	
	Type of high school attended	Governmental	348(86.8)	347(87.6)	*X*^*2 =*^ *42* *P = 0*.*654*
		Private	53(13.2)	49(12.4)	
	Participating in religious education	Yes	353(88)	367(92.3)	*X*^*2 =*^ *0*.*069* *P = 1*.*000*
		No	48(12)	29(7.3)	
	Ever discussed Sex-related matters	Yes	170(42.4)	236(59.6)	*X*^*2 =*^ *0*.*796* *P = 0*.*408*
		No	231(57.8)	160(40.4)	
**Control Group**	Sex	Male	250(61.9)	245(61.7)	*X*^*2 =*^ *0*.*58* *P = 0*.*595*
		Female	154(38.1)	152(38.3)	
	Age in year	Mean ± SD	19.67 ±126	19.69±1.17	
	Religion	Orthodox	278(68.8)	275(69.3)	*X*^*2 =*^ *9*.*49* *P = 0*.*578*
		Catholic	8(2)	6(1.5)	
		Protestant	62(15.3)	61(15.4)	
		Muslim	54(13/4)	53(13.4)	
		Others	2(0.5)	2(0.5)	
	Type of school attended	Governmental	351(86.9)	349(57.9)	*X*^*2 =*^ *1*.*19* *0*.*282*
		Private	53(13.1)	48(12.1)	
	Participating in religious education	Yes	348(86.1)	307(77.3)	*X*^*2 =*^ *1*.*44* *P = 0*.*235*
		No	56(13.9)	90(22.7)	
	Ever discussed Sex-related matters	Yes	168(41.6)	176(44.3)	*X*^*2 =*^ *0*.*457* *P = 0*.*539*
		No	236(58.4)	221(55.7)	

### Sexual and reproductive health knowledge

The finding from paired samples t-test analysis shows that there was a significant difference between the pre-exposure and post-exposure mean scores of comprehensive knowledge of condoms among respondents in the intervention group with a mean difference of 0.28 (p<0.01). No significant difference was observed in the control group with a mean of -0.05(p>0.05). Similarly, there were significant differences between the pre-exposure and post-exposure mean scores of comprehensive knowledge about contraception methods excluding condoms among respondents in the intervention group with a mean difference of 0.42(p<0.01). No significant difference was observed in the control group with a mean of 1.15 (p>0.05). However, there were significant differences between the pre-exposure and post-exposure mean scores of comprehensive knowledge on HIV/AIDS among respondents in the intervention group with a mean difference of 1.29 (p<0.01) and in the control group with a mean difference of 0.58 (p<0.01) [Table 2].

**Table 2:** Differences in Sexual & reproductive health knowledge and attitude before and after intervention among first-year students of Arba Minch University during the pre and post-exposure period, 2019.

**Variables**	**Intervention Group**		**Δ Mean** **(P-value)**	**Control Group**		**Δ Mean** **(P-value)**
	**Pre-exposure** **Mean ± SD**	**Post-exposure** **Mean ±SD**		**Pre-exposure** **Mean ± SD**	**Post-exposure** **Mean ±SD**	
Comprehensive knowledge of HIV/AIDS	3.56 ±2.13	4.86 ± 0.39	1.29, p<0.01	3.97±0.82	4.55±0.85	0.58, P<0.01
Comprehensive knowledge of contraceptive methods	3.90 ±1.75	4.32 ±1.79	0.42, p<0.01	3.96±1.67	4.11±1.01	0.15, p>0.05
Comprehensive knowledge of condoms	3.42±0.74	3.70±0.62	0.28, p<0.01	3.54±0.64	3.49±0.79	-0.05, p>0.05
Attitude toward HIV/AIDS	6.35 ±1.81	7.58 ±1.55	1.23, p<0.01	5.99 ± 1.75	6.02±1.58	0.03, p>0.05
Attitude toward condoms	24.49±5.29	25.79±7.88	1.30, p<0.01	23.80 ±6.29	27.79 ±5.48	-0.15, p>0.05
Attitudes toward gender norms	49.38 ±7.15	48.74 ±7.71	-0.64, p>0.05	49.49±8.12	49.79±6.96	0.30,p>0.05

Also finding from the independent sample t-test shows that there were significant differences between the intervention and control group mean scores of comprehensive knowledge of HIV/AIDS (mean diff. = 0.32, 95%CI = 0.22 to 0.41: p<0.01), Comprehensive knowledge of contraceptive methods (mean diff. = 0.22, 95%CI = 0.02 to 0.42: p<0.05), Comprehensive knowledge about condoms (mean diff. = 0.22, 95%CI = 0.12 to 0.32: p<0.01) [Table 3].

**Table 3:** Differences in Sexual & reproductive health knowledge and attitude after intervention among the first-year student of Arba Minch University during the pre and post-exposure period, 2019.

Subcategories	Mean ± SD		Δ Mean	[95% CI]	P-value
	Intervention Group	Control Group			
Comprehensive knowledge of HIV/AIDS	4.87±0.38	4.55 ± 0.85	0.32	*(0*.*22*,*0*.*41)*	*<0*.01*
Comprehensive knowledge of contraceptive methods	4.33±1.78	4.11±1.01	0.22	*(0*.*02*,*0*.*42)*	*<0*.05*
Comprehensive knowledge of STI	4.76 ±2.69	4.83 ±2.69	-0.08	*(-0*.*45*,*0*.*29)*	>0.05
Comprehensive knowledge of condoms	3.70±0.62	3.48±0.79	0.22	(0.12,0.32)	<0.01*
Attitude toward HIV/AIDS	7.62 ±1.54	6.06 ±1.61	1.56	*(1*.*34*,*1*.*78)*	*<0*.01*
Attitude toward condom use	25.79±7.88	23.79±5.49	2.01	*(1*.*06*,*2*.*96)*	*<0*.01*
Attitudes toward gender norms	48.85 ±7.72	49.71 ±6.94	-0.86	*(-1*.*88*,*0*.*17)*	>0.05

### Sexual and reproductive health attitude

The result of this study shows that there was a significant difference between the pre-exposure and post-exposure mean scores of attitude toward HIV/AIDS among respondents in the intervention group with a mean difference of 1.23 (p<0.001). No significant difference was observed in the control group with a mean of 0.03 (p>0.05). Similarly, there were significant differences between the pre-exposure and post-exposure mean scores of attitudes toward condoms among respondents in the intervention group with a mean difference of 1.30 (p<0.05). No significant difference was observed in the control group with a mean of -0.15 (p>0.05). However, there were no significant differences between the pre-exposure and post-exposure mean scores of Attitudes toward gender equality norms among respondents in the intervention group with a mean difference of -0.64 (p>0.05) and in the control group with a mean difference of 0.30 (p>0.05) [Table 2].

The finding from independent sample t-test shows that there were significant differences between the intervention and control group mean scores of attitude to HIV/AIDS (mean diff. = 1.56, 95%CI = 1.34 to 1.78: p<0.01) and attitude to condoms (mean diff. = 2.01, 95%CI = 1.06 to 2.96: p<0.01). However, there is no significant difference observed between the intervention and control group mean scores of attitude to gender equality norm (mean diff. = -0.86, 95%CI = -1.89 to 0.17: >0.05) [Table 3].

### Sexual behaviors among students

In the intervention group, 164(40.9%) of students at baseline and 112(28.28%) at the end line reported that they had ever practiced risky sexual behavior. In the control group, 152(37.6%) of students at baseline and 148(37.28%) at the end line reported that they had ever practiced risky sexual behavior. Also, there are statistically significant differences between the intervention and control groups in terms of changes in sexual behavior (***X***^***2***^ ***= 7*.*76; P<0*.*01)*.** Besides this 92(22.9%) of students at baseline and 102(25.8%) of students at the end line among the intervention group reported that they had ever used contraception. In the control group, 86(21.3%) of students at baseline and 89 (22.4%) at the end line reported that they had ever used contraception during their last sexual intercourse to prevent unintended pregnancy. However, there are statistically significant differences between the intervention and control groups in terms of changes in contraception utilization ***(X***^***2***^ ***= 1*.*21; P>0*.*05)*** [Tables 4 & 5].

**Table 4:** Differences in Sexual behaviors before and after intervention among first-year students of Arba Minch University during the pre and post-exposure period, 2019.

Variables	Intervention Group		(P-value)	Control Group		(P-value)
	Pre-exposure N (%)	Post-exposure N (%)		Pre-exposure N (%)	Post-exposure N (%)	
Risky sexual behavior	164(40.9)	112(28.3)	< 0.01*	152(37.6)	148(37.3)	>0.05
Contraception utilization	92(22.9)	102(25.8)	>0.05	86(21.3)	89(22.4)	>0.05
Intention to use condoms	148(36.9)	162(40.9)	>0.05	112(27.7)	120(30.20)	>0.05
The magnitude of self-reported unintended pregnancy	48(12.0)	25(6.31)	>0.51	40(9.9)	47(11.8)	>0.05
Consistent and correct use of condoms	59(14.7)	64(16.2)	0.01*	55(13.6)	58(14.6)	>0.05
Decision-making toward contraception utilization	70(17.5)	73(18.4)	*<0*.01*	69(17.1)	68(17.1)	*<0*.01*
Ever screen for HIV	152(37.9)	199(49.6)	*< 0*.01*	135(33.4)	175 (43.3)	>0.05

**Table 5:** Differences in sexual behaviors among first-year students of Arba Minch University during the pre and post-exposure period, 2018/19.

Variables	Pre-intervention		P-value	Post-intervention		P-value
	Intervention Group N (%)	Control Group N (%)		Intervention Group N (%)	Control Group N (%)	
Risky sexual behavior	164(40.9)	152(37.6)	X^2^ = 0.91 P>0.05	112(28.28)	148(37.28)	***X***^***2***^ ***= 7*.*76*** *P<0*.01*
Ever screen for HIV	152(37.9)	135(33.4)	X^2^ = 1.77 P>0.05	195(49.7)	172 (43.3)	X^2^ = 2.77 P>0.05
Consistent and correct use of condoms	59(14.7)	55(13.6)	X^2^ = 0.20 P>0.05	64(16.2)	58(14.6)	X^2^ = 0.37 P = 0.56
Intention to use condoms	115(28.67)	109(26.98)	X^2^ = 0.20 P>0.05	162(40.9)	120(30.20)	***X***^***2***^ ***= 9*.*87*** *P<0*.05*
Decision-making toward contraception utilization	70(17.5)	69(17.1)	X^2^ = 0.02 P>0.05	73(18.4)	68(17.1)	X^2^ = 0.231 P>0.05
Contraception utilization	92(22.9)	86(21.3)	X^2^ = 0.32 P>0.05	102(25.8)	89(22.4)	X^2^ = 1.21 P>0.05

### Effect of curriculum-based sexual and reproductive health education on students’ knowledge and attitude

An inverse probability weighting analysis was conducted to see the effect of curriculum-based sexual and reproductive health education on students’ knowledge and attitude. All outcome variables were weighted by the baseline characteristics of study participants (sex, age, residence, religion, attendance of religious education, and type of school they attended) to reduce the effect of selection bias. The finding of this study shows that in the intervention group, the student’s average change of comprehensive HIV/AIDS knowledge score was 0.22 higher than the average score of the students in the control group (ATE = 0.22,95% CI, 0.14 to 0.29; p < 0.01). The average change of attitude toward the HIV/AIDS score of the students in the intervention group was 1.32 higher than the average change score of students in the control group (ATE = 1.32, 95% CI, 1.18 to 1.47; p < 0.01). In the intervention group, the student’s average change of comprehensive contraceptive methods knowledge score was 0.22 higher than the average score of students in the control group (95% CI, 0.01 to 0.43; p > 0.05). Moreover, the finding of this study reveals that comprehensive sexual and reproductive health intervention has a significant effect in reducing risky sexual behavior among intervention groups with an average change of -0.05 with (ATE = 0.05, 95% CI, -0.08 to -0.01; p < 0.05). However, this finding shows that there is no significant effect on the consistent and correct use of condoms with (ATE = 0.02, 95% CI, -0.03 to 0.07; p > 0.05) and contraceptive utilization with (ATE = 0.04, 95% CI, -0.03 to 0.09; p < 0.05) [Table 6].

**Table 6:** Effect of curriculum-based sexual and reproductive health education on students’ knowledge and attitude among first-year Arba Minch University students, Arba Minch, Ethiopia, 2019.

Variables	Regression coefficient	95% CI	p-value
Comprehensive knowledge of HIV/AIDS	0.31	(0.22,0.40)	***< 0*.*01*** *
Comprehensive knowledge of contraceptive methods	0.22	(0.01,0.43)	***<0*.*037*** *
Comprehensive knowledge of STI	0.01	(-0.36, 3.76)	>0.05
Comprehensive knowledge of condoms	0.23	(0.13,0.33)	***< 0*.*001*** *
Attitude toward HIV/AIDS	1.54	(1.31,1.77)	***< 0*.*01*** *
Attitude toward condoms	1.83	(1.19,2.77)	***< 0*.*01*** *
Risky sexual behavior	-0.05	(-0.08,-0.01)	***<0*.*05*** *
Ever screen for HIV	0.66	(-0.04,0.14)	>0.05
Consistent and correct use of condoms	0.02	(-0.03,0.07)	>0.05
Intention to use condoms	0.11	(0.04,0.18)	***<0*.*01*** *
Contraception utilization	0.04	(-0.03,0.09)	>0.05

* P < 0.05 sig. (2-sided).

## Discussion

This study was conducted to assess the effectiveness of curriculum-based comprehensive sexual and reproductive health education at Arba Minch University. The finding of this study shows that after intervention students who practice sexual behavior reduced significantly. Besides this, after this education, there is a significant difference between intervention and control concerning sexual and reproductive health knowledge and attitude. However, there is no significant difference between intervention and control groups in the changes in scores concerning HIV testing and contraception utilization among Arba Minch university students.

Curriculum-based sexual and reproductive health education significantly reduced risky sexual behaviors among intervention groups compared to the control group. This finding is in line with studies conducted in various developing countries [32,33]. They may give direction to strengthen the implementation of this education to help young people practice risky sexual behavior. Because most young people practice sexual activity, it may have been valuable to explore other behaviors related to sexual and reproductive health that are more relevant to this age group. This finding is important for policy to improve and strengthen implementation of curriculum-based sexual and reproductive health education to prove age-appropriate information regarding dispelling fears and anxiety in dealing with puberty changes, recognition of SRH risks, and how they can be avoided [34].

This curriculum-based sexual and reproductive health education brings differences in the changing students’ consistent and correct use of condoms among the intervention group, but not in the control group. This finding was supported by a cluster randomized controlled trial study conducted in Tanzania [35]. This finding is also supported by peer sexual health education intervention implemented in Zambian secondary schools [21]. Another finding from a study conducted in Mexican among high school students also supported our finding [36]. This is promising in the light of findings that may give direction providing sexual and reproductive health education may help to maintain sexual and reproductive health, youths need access to accurate information and the safe, effective, affordable, and acceptable contraception method of their choice [32].

Providing curriculum-based sexual and reproductive health education interventions has a significant improvement in sexual and reproductive knowledge. This finding is supported by a quasi-experimental study conducted in Northern Ghana and Uganda [34,37]. The systematically reviewed study also supports this finding [32]. This finding gives hint strengthened curriculum-based sexual and reproductive health education intervention may help to improve knowledge which is the major problem of Ethiopia’s young people who lack comprehensive SRH knowledge, which has been reported in the different studies conducted in Ethiopia [22,38]. Although knowledge necessary prerequisite to getting behavior change which is a promise to prevent young people, who are just beginning to be exposed to SRH risks [2].

On the other hand, the finding of this study shows that curriculum-based sexual and reproductive health education has a significant effect on the increase in the mean scores of sexual and reproductive health attitudes. This finding is in line with a study conducted in Tanzania, and Southwest China [35,39]. This might be because most university students are at this age with a very high risk of sexually deviant behaviors because they have physically experienced sexual maturity, have psychologically attracted to the opposite sex, and have had fluctuating emotions, so they require reproduction health information which is accurate and complete, based on the development, and from the right source. The readers should consider limitations like not being confident to conclude the long-term effect of CBSRHE since the intervention period was six months. Even though we tried to match some confounding factors such as social–demographic characteristics, there may be some unknown factors influencing the effect of the intervention, which might increase or decrease the real effect of an intervention. However the study participants were young and the study included a sensitive question related to sexual behaviors, data were collected using self-administer questionnaires which might be subject to response bias, and as the study was first that may help to decide on the program. The experimental nature of this study is very significant to reach a on decision to the effectiveness of CBSRHE. But the readers should consider limitations like response bias as the data was collected with self administer questionnaires and this study may not show the long term impact of CBSRHE.

## Conclusion

In conclusion, this study provides insights regarding the short-term effect of CBSRHE on the knowledge and attitude on the risky sexual behaviors among Arba Minch University year one student. It was found that there was a significant difference in knowledge and attitude on risky sexual behaviors among the students in the two arms. Therefore, the Ministry of education should adopt and integrate the course into all universities in the country and further comparative studies must be done to determine the long-term effect of the course supported with models and/or theories like the theory of change.

## Supporting information

S1 File (DOCX)

S1 Data (SAV)
