# Supplementary material for: Effectiveness of curriculum-based sexual and reproductive health education on healthy sexual behaviors among year one students at Arba Minch University: A quasi-experimental study
Source: PLoS One. 2023 Oct 31;18(10):e0288582. doi: 10.1371/journal.pone.0288582 (PMC10617698; doi:10.1371/journal.pone.0288582)
Supplement: S1 File — (DOCX) [file pone.0288582.s001.docx]

**Annexe A: Data collection tools for quantitative study**

**Questionnaire for data collection on effect of comprehensive sexual and reproductive health education interventions among first year students in Arba Minch University: A Quasi-Experimental study**

Code-------------

1. **Socio-Demographic Variables**

| Questions | | Response Category | | Remark |
| --- | --- | --- | --- | --- |
| 101 | Sex | 1. Male 2. Female | |  |
| 102 | Age | ____________ years | |  |
| 103 | Residence | 1. Urban 2. Rural | |  |
| 104 | From which region you come to Arba Minch University  *(Please write your Region)* | Specify______________________ | |  |
| 105 | Religion | 1. Muslim  2.Orthodox  3.Protestant | 4.Catholic  5.Other specify________ |  |
| 106 | Campus | 1. Main campus  2.Nachsar campus  3.Abaya campus | 4.Chamo campus  5. Kulfo campus  6.Sawla campus |  |
| 107 | Are you participating in religious education or services? | 1. Yes | 2.No |  |
| 108 | What type of school you attended? | 1.Govermental | 2.Private |  |
| 109 | History of Substance Use | 1. Yes | 2.No |  |
| 110 | Have you ever discussed sex related issue with your parents? | 1. Yes | 2.No |  |

1. **knowledge of sexual and reproductive health**

| ***Please read question carefully and give the marks based on their importance*** | |  |
| --- | --- | --- |
| ***201*** | Have you heard about puberty? | 1. Yes 2. No ***(skip to Q 2.4)*** |
| 202 | What are the sources of information about puberty?  ***(you can choose more than one options)*** | 1. teacher 2. Mother 3. Father 4. Brother 5. Sister 6. Other family members 7. Friends 8. Health professionals 9. Media 10. Other, specify,___ |
| 203 | Do you know about sexual and reproductive systems of men and women? | 1. Yes 2. No ***(skip to Q 2.5)*** |
| 204 | What are the source of information about sexual and reproductive systems of men and women?  ***(you can choose more than one options)*** | 1. Teacher 2. Mother 3. Father 4. Brother 5. Sister 6. Other family members 7. Friends 8. Health professionals 9. Media 10. Other, specify________ |
| 205 | What are the sources of information to begin friendship relation?  ***(you can choose more than one options)*** | 1. Teacher 2. Mother 3. Father 4. Brother 5. Sister 6. Other family members 7. Friends 8. Health Professionals 9. Media 10. Other, specify |
| 1. **Risk Sexual Related Variables**   The purpose of this section is to establish the types of relationships that young people from their degree of sexual intimacy and communication, and the possible attendant risks of unwanted pregnancy or sexually transmitted infectious (STIs).   \| 301 \| \| Have you ever had sexual intercourse in the last 12 months? \| 1. Yes 2. No***(skip to Q-3.29)*** \| \| --- \| --- \| --- \| --- \| \| 302 \| \| Would you say it was? \| 1. planned 2. unplanned \| \| 303 \| \| Think back to the first time you had sex with your friend, Would you say. \| 1. I forced my frind to have intercourse against her/his will 2. I persuaded my frind to have intercourse 3. my frind persuaded me to have intercourse 4. my friend forced me to have intercourse 5. We were both equally willing \| \| 304 \| \| How old are you at the time when you start your first sexual relationship? \| -----------------Years \| \| 305 \| \| How about your partner’s age when you start your first sexual relationship? \| -----------------Years \| \| 306 \| \| What was your reason to start sexual intercourse? \| 1. In a marriage 2. Peer pressure 3. To academic benefit(for grade) 4. For financial purpose(to get money) 5. Other, Specify— \| \| 307 \| \| With whom 1^st^ sex was made? \| 1. Boyfriend/girlfriend 2. Unfamiliar person 3. Teacher 4. Stranger 5. Commercial Sex Workers(only for male students) 6. Other, Specify------------- \| \|  \| \| \| \| \| 308 \| How many months between the time you started your relationship and the time you first had sex with your friend? \| \| ------------------------Months \| \| 309 \| When you started your sexual relationship, was your freind? \| \| 1. Single 2. Married 3. Divorce 4. Widowed 5. I don’t know \| \| 310 \| When you started you relationship with your freind was your freind? \| \| 1. Full time student 2. Worker/Employee 3. Merchant 4. Other, specify,________ 5. I am not sure \| \| 311 \| How would you describe your sexual relationship with your friend? Was (is) it \| \| 1. Casual friendship 2. Serious relationship but with no intention of marriage 3. Important/might lead to marriage 4. Engaged to be married 5. I am not sure \| \| 312 \| How do you think your sexual ferind would describe her/ his relationship to you? \| \| 1. as a casual friendship 2. a serious relationship but with no intention of marriage 3. an important relationship that might lead to marriage 4. Engaged to be married 5. I am not sure \| \| 313 \| During first sexual intercourse with your friend , did you used any method to avoid a pregnancy? \| \| 1. Yes 2. No ***(skip to Q-3.20)*** \| \| 314 \| What method did you and your friend used?  ***(You can choose more than one option)*** \| \| 1. Condom 2. Birth control pill 3. Depo-Provera( inject able) 4. Withdrawal 5. Calendar Method Other, specify………… \| \| 315 \| Did you ever discuss about avoiding pregnancywith your Friend? \| \| 1. Yes 2. No ***(Skip to 3.20)*** \| \| 316 \| If yes, did you discuss avoiding pregnancybefore or after you had intercourse? \| \| 1. Before first intercourse 2. After first intercourse 3. Never \| \|  \| \| \| \|  \| 317 \| Who decided to use contraception method? \| \| 1. My decision 2. My sexual friend’s decision 3. Joint decision \| \| \| --- \| --- \| --- \| --- \| --- \| \| 318 \| *Other than the first time sexual patner,* did you had sex with another girl/boy in last 12 months? \| \| 1. Yes 2. No ***(skip to Q3.22)*** \| \| \| 319 \| *If yes,* did you and your friend ever use any method to avoid pregnancy? \| \| 1. Yes 2. No \| \| \| 320 \| In whole life with how many people you had sexual intercourese ? \| \| 1. One person 2. Two person 3. Three or more person \| \| \| 321 \| After you join AMU with how many people you had sexual intercourse? \| \| 1. One person 2. Two person 3. Three or more person \| \| \| 322 \| Have you ever used condom in last 12 months? \| \| 1. Yes 2. No ***(Skip to Q 3.27)*** \| \| \| 323 \| How often did you use condom during sexual intercourse in your life? \| \| 1. Sometimes 2. Occasionally 3. Consistently \| \| \| 324 \| Did you use condom the last time you had sexual intercourse? \| \| 1. Yes ***(Skip to Q 3.28*** ) 2. No \| \| \| 325 \| What was your reason(s) to not using condom?  ***(You can choose more than one option)*** \| \| 1. Dislike of condom 2. I always used condom 3. I have trusted my partner 4. Reduces sexual pleasure 5. Embarrassed to buy 6. Other, Specify ------------- \| \| \| 326 \| Have you ever have sex with Commercial Sex Workers***?( only for male students)*** \| \| 1. Yes 2. No \| \| \| 327 \| Have you ever chewing khat? \| \| 1. Yes 2. No \| \| \| 328 \| Have you ever drink alcohol? \| \| 1. Yes 2. No \| \| \| 329 \| Have you ever smoke tobacco? \| \| 1. Yes 2. No \| \| \| 330 \| Have you ever smoke illicit drugs (Ganja Heroni, Hashish) etc? \| \| 1. Yes 2. No \| \| \|  \| \| \| \| \| \| 331 \| \| Have you ever seen pornographic (Sex film) movie? \| \| 1. Yes 2. No \| \| 332 \| \| Have you ever attending at night club? \| \| 1. Yes 2. No \| \| 333 \| \| Did you have any discussion with your parent, or closer friend regarding sexual matters? \| \| 1. Yes 2. No***(Skip to Q 3.37)*** \| \| 334 \| \| How often did you discussion with your parents or closer friend regarding sexual matters? \| \| - 1. Sometimes   2. Occasionally   3. Consistently \| \| 335 \| \| Have you ever been pregnant ?( **female only)** \| \| 1. Yes 2. No ***(skip to 4.1)*** \| \| 336 \| \| Did this pregnancy? \| \| 1. Wanted 2. Unwanted \| \| 337 \| \| What happened to the (last) pregnancy? \| \| 1. Currently pregnant 2. Aborted 3. Miscarriage 4. Live-birth 5. Not sure \|   **THIS PAGE IS ONLY FOR THOSE WHO HAVE NEVEREXPERIENCED SEXUAL INTERCOURSEIN AMU**   \| - ***Please read the question carefully and give your best reason among the choice*** \| \| \| \| --- \| --- \| --- \| \| 338 \| What is the reason for not having intercourse after you join AMU?***(you can choose more than one options)*** \| 1. I don't feel ready to have sex 2. I have not had the opportunity. 3. I think that sex before marriage is wrong 4. I am afraid of getting pregnant 5. I am afraid of getting HIV/AIDS or another sexually transmitted infection. \| \| 339 \| When do you plan to have sexual intercourse?  ***Single answer only*** \| 1. I plan to wait until marriage 2. I plan to wait until I am engaged to be married 3. I plan to wait until I find someone I love 4. I plan to have sexual intercourse when an opportunity comes along \| \| 340 \| Do you feel any pressure from others to have sexual intercourse? \| 1. Yes 2. No***(skip to 5.1)*** \| \| 341 \| If yes, from whom do you feel pressure? \| 1. Friends 2. Relatives 3. Teachers 4. Unfamiliar people 5. Other, specify,_____________ \|  1. **HIV/AIDS and sexually transmitted diseases related Variables**  \| ***Dear student hear below there are some question that ask you knowledge about HIV/AIDS and sexually transmitted diseases .so read it carefully and choice your best answer*** \| \| \| \| --- \| --- \| --- \| \| 401 \| Have you heard of HIV or AIDS ? \| 1. Yes 2. No***(Skip to Q 5.14)*** \| \| 402 \| Is it possible to cure AIDS? \| 1. Yes 2. No \| \| 403 \| Do you think that a person with HIV always looks unhealthy ? \| 1. Yes 2. No \| \| 404 \| Do you know how to obtain testing and counseling to determine HIV status? \| 1. Yes 2. No ***(Skip to Q 5.7)*** \| \| 405 \| If yes have you ever tested and counseled to determine HIV status? \| 1. Yes 2. No \| \| 406 \| If yes what is your HIV status? \| 1. Positive 2. Negative 3. Unknown \| \| 407 \| Do you know about route of transmission of HIV/AID?  ***(you can choose more than one options)*** \| 1. HIV can be transmitted by sexual intercourse 2. HIV can be transmitted from mother to child 3. HIV can be transmitted by sharing needle or syringe 4. HIV can be transmitted by shaking hand 5. HIV can be transmitted by eating and drinking from the same plate or glass of an HIV-positive 6. HIV can be transmitted by using public toile 7. HIV can be transmitted through mosquito bit \| \| 408 \| Do you know about prevention and control of HIV/AID***?***  ***(You can choose more than one options)*** \| 1. HIV can be prevented by Avoid multiple sexual partners 2. HIV can be prevented by not sharing needle or syringe 3. HIV can be prevented by properly using condom during sexual intercourse 4. HIV transmission can be avoided by remaining faithful to a single partner 5. HIV transmission can be avoided by a blood test before marriage \|  \| 409 \| If one of your relative, who is HIV positive, becomes ill, would you agree to care for her/him in your house? \| 1. Agree 2. Disagree 3. I don’t known \| \| --- \| --- \| --- \| \| 410 \| If your friend is HIV positive, would you agree to continue your friendship with him/her? \| 1. Agree 2. Disagree 3. I don’t known \| \| 411 \| If a shopkeeper or food seller is HIV positive, would you agree to buy items from him/her? \| 1. Agree 2. Disagree 3. I don’t known \| \| 412 \| If a student is HIV positive, would you agree allowed to continue his/her studying in school? \| 1. Agree 2. Disagree 3. I don’t known \| \| 413 \| If a teacher is HIV positive, would you agree allowed to continue his/her teaching in school? \| 1. Agree 2. Disagree 3. I don’t known \| \| 414 \| Have you ever heard of sexually transmitted infections? \| 1. Yes 2. No ***(Skip to Q 6.1)*** \| \| 415 \| ***If yes , what are they?***  ***(You can choose more than one options)*** \| 1. Gonorrhea 2. Syphilis 3. AIDS 4. Trichomonas vaginalis 5. Candidiasis 6. Others(specify)--------------- \| \| 416 \| What are the signs and symptoms of a sexually transmitted disease in a man?  ***(You can choose more than one options)*** \| 1. Discharge from penis 2. Pain during urination 3. Ulcers/sores in genital area 4. Lower abdomen pain 5. Itchy perineum 6. Other (specify)…………… \| \| 417 \| What are the signs or symptoms of a sexually transmitted disease in a women?  ***(You can choose more than one options)*** \| 1. Vaginal discharge 2. Pain during urination 3. Ulcers/sores in genital area 4. Lower abdomen pain 5. Itchy perineum 6. Other (specify)…………… \| | | |

| 418 | If a friend of yours needed treatment for a sexually transmitted disease, where could he or she obtain such treatment?  ***(You can choose more than one options)*** | 1. Shop 2. Pharmacy 3. Govt. hospital/health center 4. Private clinic 5. Other (SPECIFY)………… |
| --- | --- | --- |
| 419 | Have you ever had a sexually ***transmitted disease?*** | 1. Yes 2. No***(Skip to Q 6.1)*** |
| 420 | (On the last occasion) did you seek treatment? | 1. Yes 2. No |
| 421 | Where did you seek treatment? | 1. Shop 2. Pharmacy 3. Govt. hospital/health center 4. Private clinic 5. Other …………………… |
| 422 | Did your sexual partner also obtain treatment? | 1. Yes 2. No 3. Don't know |

1. **Family planning Related Variables**

| **Here are some questions pleases read those questions carefully and choice the best choice one** | | | | | |
| --- | --- | --- | --- | --- | --- |
| 501 | Have you heard about contraceptive methods? | | | 1. Yes 2. No ***(Skip to Q 7.1)*** | |
| 502 | If yes what are they  ***(You can choose more than one options)*** | | | 1. Oral contraceptive pill 2. Depo(injections) 3. Emergency contraceptive pill 4. Implant 5. IUCD 6. Male Condom 7. Female condom 8. Calendar method 9. Others,(Specify), Specify,_________________ | |
| 503 | Sources of information about contraceptive methods  ***(You can choose more than one options)*** | | | 1. Media 2. Formal education 3. Parent 4. Health institutions 5. Friends 6. University clubs 7. Other sources, specify,_____________ | |
| 504 | Which one of the following method of contraceptive used to prevent STI? | | | 1. Oral contraceptive pill 2. Depo 3. emergency contraceptive pill 4. Implant 5. IUCD 6. Condom 7. Other, specify,________ | |
| 505 | Did you know side effects of family planning methods? | | | 1. Yes 2. No***(skip to 6.7)*** | |
| 506 | ***If yes,*** what are they  ***(You can choose more than one options)*** | | | 1. Vaginal Bleeding 2. Breast pain 3. Stop menstruation 4. Irregular menstruation 5. Other(**Specify),_____________** | |
| 507 | Did you know any place or person where young people could obtain this contraceptive method?  ***(You can choose more than one options)*** | | | 1. Shop 2. Pharmacy 3. Health Centre/Hospital 4. Private Clinic 5. Friend 6. Other **(Specify) …** | |
| 508 | Which method do you think is most suitable for young people?***Circle one answer*** | | | 1. Oral contraceptive pill 2. Depo(injections) 3. Emergency contraceptive pill 4. Implant 5. IUCD 6. Male Condom 7. Female condom 8. Calendar method 9. Others,**(Specify),___________** | |
| 509 | Have you ever used anything to delay or avoid getting pregnancy | | | 1. Yes 2. No ***(skip to 7.1)*** | |
|  | | | | | |
| 1. 51   510 | | ***If yes,*** which method did you used?  (You can choose more than one options) | 1. Oral contraceptive pill 2. Depo(injections) 3. Emergency contraceptive pill 4. Implant 5. IUCD 6. Male Condom 7. Female condom 8. Calendar method 9. Others,(Specify),____________ | |  |
| 511 | | ***If yes,*** from where did you obtain this method | 1. Hospital 2. Health center 3. Health post 4. Student clinic 5. Drug store/pharmacy 6. other specifies---------------- | |  |

1. **Condom Related Variables**

| 601 | Do you know male condom? | 1. Yes 2. No | | |
| --- | --- | --- | --- | --- |
| 602 | Do you know female condom? | 1. Yes 2. No | | |
| 603 | ***If yes,*** from where you obtain condoms?  ***(You can choose more than one options)*** | 1. Student clinic 2. Private pharmacy 3. Health center 4. Hospital 5. Friends | | |
| 604 | Do you know how to use a condom correctly | 1. Yes 2. No ***(skip to 7.7)*** | | |
| 605 | ***If yes,***have you ever recived any information on how to use a condom correctly? | 1. Yes 2. No | | |
| 606 | What are source of information on how to use a condom correctly?  ***(You can choose more than one options)*** | 1. Health profession 2. Peer /friend 3. Media/TV/radio 4. school 5. immediate Family 6. Other (specify) …………………… | | |
| 607 | Have you or a partner ever used a condom Consistent in the last 12 months? | 1. Yes 2. No***(skip to 7.9)*** | | |
| 608 | ***If yes,***have you ever experienced a condom that split or broke during intercourse? | 1. Yes 2. No | | |
|  | | | | |
| **Attitudes towards condom** | | | | |
| ***People have different opinions about condoms. please read out some opinions. For each one, please write whether you agree or disagree, or whether you don't know*** | | Agree | Don’t Known/  not sure | Disagree |
| 609 | Condoms are an effective method of preventing pregnancy | 1 | 2 | 3 |
| 610 | Condoms can be used more than once | 1 | 2 | 3 |
| 611 | A girl can suggest to her boyfriend that he use a condom | 1 | 2 | 3 |
| 612 | A boy can suggest to his girlfriend that he use a condom | 1 | 2 | 3 |
| 613 | Condoms are an effective way of protecting against HIV/AIDS | 1 | 2 | 3 |
| 614 | Condoms are suitable for casual relationships | 1 | 2 | 3 |
| 615 | Condoms are suitable for steady, loving relationships | 1 | 2 | 3 |
| 616 | It would be too embarrassing for someone like me to buy or obtain condoms | 1 | 2 | 3 |
| 617 | If a girl suggested using condoms to her partner, it would mean that she didn't trust him | 1 | 2 | 3 |
| 618 | Condoms reduce sexual pleasure | 1 | 2 | 3 |
| 619 | Condoms can slip off the man and disappear inside the woman's body | 1 | 2 | 3 |
| 620 | If unmarried couples want to have sexual intercourse before marriage, they should use condoms | 1 | 2 | 3 |
| 621 | Condoms are an effective way of protecting against sexually transmitted diseases | 1 | 2 | 3 |

1. **Gender Related Variables**

| ***People have different opinions about gender. please read out some opinions. For each one, please write whether you agree or disagree, or whether you don't know*** | | Agree | Don't know/not sure | Disagree |
| --- | --- | --- | --- | --- |
| 701 | Do you believe it's all right for unmarried boys and girls to have sexual partner? | 1 | 2 | 3 |
| 702 | Do you believe it's all right for boys and girls to kiss and touch each other | 1 | 2 | 3 |
| 703 | Do you believe there is nothing wrong with unmarried boys and girls having sexual intercourse if they love each other. | 1 | 2 | 3 |
| 704 | Do you think that sometimes a boy has to force a girl to have sex if he loves her. | 1 | 2 | 3 |
| 705 | Do you belive a boy will not respect a girl who agrees to have sex with him. | 1 | 2 | 3 |
| 706 | Do you belive most girls who have sex before marriage regret it afterwards. | 1 | 2 | 3 |
| 707 | Do you belive most boys who have sex before marriage regret it afterwards. | 1 | 2 | 3 |
| 708 | Do you belive a boy and a girl should have sex before they become married | 1 | 2 | 3 |
| 709 | Do you belive that girls should remain *virgins* until they marry. | 1 | 2 | 3 |
| 710 | Do you belive that boys should remain *virgins* until they marry. | 1 | 2 | 3 |
| 711 | Do you belive it is sometimes justifiable for a boy to hit his girlfriend. | 1 | 2 | 3 |
| 712 | Do you belive that most of my friends think that *sex for money* is good | 1 | 2 | 3 |
| 713 | Do you belive It's all right for boys and girls to have sex with each other provided that they use methods to stop pregnancy. | 1 | 2 | 3 |
| 714 | Do you belive that most of ur friends who have sex with someone use condoms regularly. | 1 | 2 | 3 |
| 715 | Do you belive that you should have confident that you can insist on condom use every time you have sex. | 1 | 2 | 3 |
| 716 | Do you belive you would never consider having an abortion on your self or for ur partner. | 1 | 2 | 3 |
| 717 | Do you belive that it is mainly the woman's responsibility to ensure that contraception is used regularly. | 1 | 2 | 3 |
| 718 | Do you belive that you should be in love with someone before having sex with them. | 1 | 2 | 3 |
| 719 | Do you belive that you feel that you now how to use a condom properly. | 1 | 2 | 3 |
| 720 | Do you belive most of your friends would never consider having an abortion for themselves or their partner | 1 | 2 | 3 |
| 721 | Do you belive that men need sex more frequently than do women | 1 | 2 | 3 |
| 722 | Do you belive that most of your friends believe that you should be in love before you have sex with someone. | 1 | 2 | 3 |
| 723 | Do you belive that you would refuse to have sex with someone who is not prepared to use a condom. | 1 | 2 | 3 |
| 724 | Do you belive that One night stands are OK | 1 | 2 | 3 |

THANKS FOR YOUR COOPERATION!!!!!
